# Recurrent Venous Thromboembolism in a Patient with Klippel-Trenaunay Syndrome Despite Adequate Anticoagulation with Warfarin

**DOI:** 10.7759/cureus.7576

**Published:** 2020-04-07

**Authors:** Zekarias T Asnake, Troy J Fishman, Liang Sun, Joshua K Salabei

**Affiliations:** 1 Internal Medicine, University of Central Florida College of Medicine, Hospital Corporation of America (HCA) North Florida Division, Gainesville, USA; 2 Internal Medicine, University of Central Florida College of Medicine · Hospital Corporation of America (HCA) North Florida Division, Gainesville, USA

**Keywords:** klippel-trenaunay syndrome, pulmonary embolism, varicose veins, venous thromboembolism, prophylaxis, anticoagulation

## Abstract

Klippel-Trenaunay syndrome (KTS) is a rare genetic condition defined by capillary malformation, venous malformation, and soft tissue and bony overgrowth. Due to venous malformations, individuals are predisposed to intravascular coagulopathy leading to thrombosis and thromboembolism. However, anticoagulating these patients long-term remains a challenge because of the presence of capillary malformations that increase bleeding risk. We present a rare case of a 30-year-old Caucasian male with KTS and history of gastrointestinal bleeding who has been on anticoagulation since the age of 7 and has had three different inferior vena cava filters placed during his lifetime. At presentation, he had dyspnea with stable vital signs. His prothrombin time/international normalized ratio was 37.3 and 3.2, respectively and chest computed tomography showed bilateral segmental pulmonary embolism (PE). He was treated with heparin drip and his home anticoagulation was switched from warfarin to apixaban at the time of discharge for better anticoagulation optimization. KTS is a condition associated with venous thromboembolic complications that can be difficult to manage. PE should remain on the top of the list of differential diagnoses in patients with KTS presenting with dyspnea even if laboratory findings suggest an alternate diagnosis.

## Introduction

Klippel-Trenaunay syndrome (KTS; also known as angio-osteohypertrophy syndrome, congenital dysplastic angiopathy, and Klippel-Trenaunay-Weber syndrome) is a rare genetic condition that affects about 1 in 100,000 individuals and has been defined as a triad of capillary malformation, venous malformation, and limb and soft tissue overgrowth beginning in infancy [1]. Most genomically analyzed cases of KTS have been shown to be caused by mosaic-activating mutations in the PIK3CA gene that encodes the p110 alpha (p110α) protein, a subunit of the enzyme phosphatidylinositol 3-kinase (PI3K). Abnormally active PI3K allows cells to grow and divide continuously leading to increased cell proliferation and abnormal growth of bones, soft tissues, and blood vessels [2,3]. With this overgrowth, venous malformations remain a major feature of KTS. Such malformations, especially of deep veins, increase the risk for venous thromboembolism (VTE) [4]. It is well established that the incidence of VTE in KTS is between 14 and 22% which is equal to or higher than the incidence associated with most other risk factors for VTE [5].

Currently, there are no established guidelines on anticoagulation prophylaxis for patients with KTS. Also, anticoagulation in these patients is further complicated because of their increased risk for bleeding owing to the increased prevalence of arteriovenous malformation (AVM) [6]. We present the case of a 30-year-old male who was diagnosed with KTS in utero. He is known to have recurrent VTE despite laboratory evidence of achieving prophylactic anticoagulation with warfarin. In this case, we have highlighted the following: (1) The importance of having VTE at the top of the list of differential diagnoses in patients with KTS even when laboratory evidence may show otherwise, (2) the dilemma that clinicians can face when optimizing anticoagulation in patients with KTS, and (3) the importance of developing publicly available guidance on preventing/managing recurrent VTE in patients with KTS.

## Case presentation

A 30-year-old Caucasian male with a history of KTS diagnosed in utero presented with sudden onset chest pain, lightheadedness, presyncope, and shortness of breath. Routine laboratory tests and imaging, including D-dimer levels and computer tomography angiogram respectively, showed subtle small bilateral peripheral filling defects within the pulmonary branches suggesting bilateral PE despite being adequately anticoagulated with warfarin. His prothrombin time (PT)/international normalized ratio (INR) on presentation was 37.3/3.2 (Table 1).

**Table 1:** Pertinent laboratory data at the time of presentation PT: Prothrombin time; INR: International normalized ratio.

Complete blood count	Levels on admission	Normal range
White blood cells	6.6	(4.5-11.0 x 10^3^/mm^3^)
Red blood cells	3.12	(3.80-5.20 million /uL)
Hemoglobin	7.8	(12.0-15.0 g/dL)
Platelet Count	281	(150-450 x 10^3^/mm^3^)
Basic Metabolic Profile		
Sodium	144	(136-145 mmol/L)
Potassium	3.6	(3.5-5.1 mmol/L)
Blood urea nitrogen	17	(7-18 mg/dL)
Creatinine	0.84	(0.60-1.30 mg/dL)
Glucose	99	(74-106 mg/dL)
Calcium	8.5	(8.5-10.1 mg/dL)
Aspartate aminotransferase	54	(15-37 Units/L)
Alanine aminotransferase	199	(12-78 Units/L)
Alkaline phosphatase	70	(46-116 Units/L)
Coagulation profile		
PT	37.3	(10.2-12.9 seconds)
INR	3.2	(0.9-1.1)
Activated partial thromboplastin time	38	(25.1-36.5 seconds)
D-dimer	3.72	(<0.5 ng/L)

**Figure 1:**
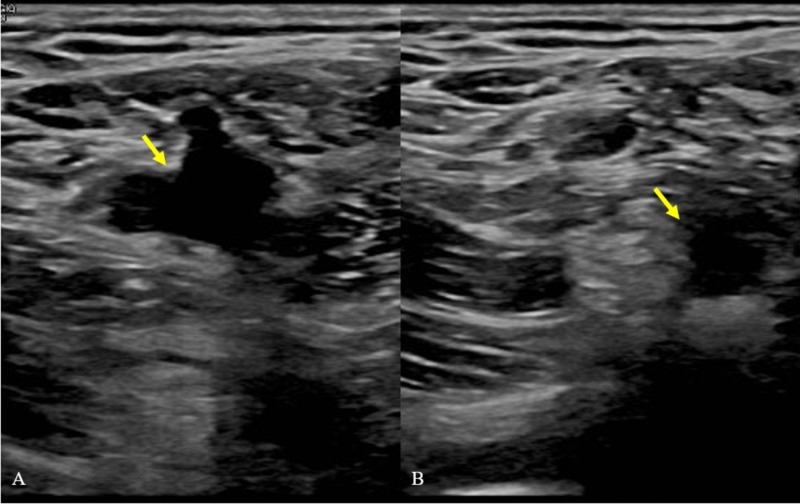
Duplex venous ultrasound of the left lower femoral vein (A) Representative image of the non-compressed femoral vein. The yellow arrow shows an uncompressed patent femoral vein. (B) Lack of complete collapse upon compression is indicative of venous thromboembolism. The yellow arrow shows the partially compressed femoral vein.

**Figure 2:**
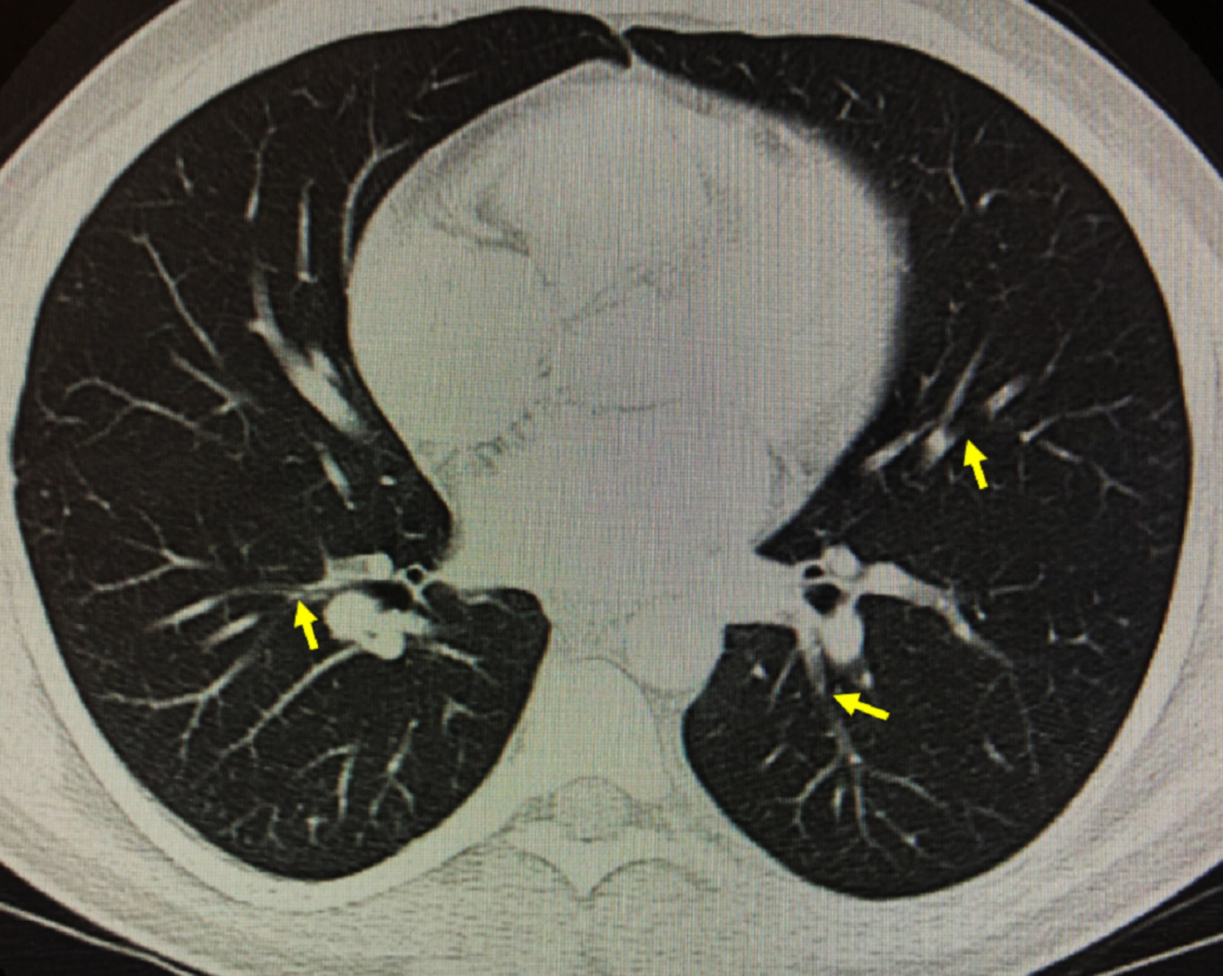
Computed tomography angiography of the chest Cross-sectional representative image of the chest. Notable subtle small bilateral filling defects within the pulmonary branches are indicated with the yellow arrows.

His blood pressure was 132/74 mmHg, pulse was 69 beats per minute, respiratory rate was 17 breaths per minute, and SPO_2_ on room air was 98%. Physical examination revealed a clear chest with good air entry bilaterally. Hypertrophy of his bilateral lower extremities was noted on physical exam as well. Nevus flammeus was noted over the lateral aspect of the left dorsal feet and the lateral aspect of the left knee and thigh. These skin findings were non-tender, non-erythematous, and showed no other signs of cellulitis. Venous ultrasound of his bilateral lower extremity was significant for deep venous thrombosis (DVT) (Figure 1). Computed tomography angiography (CTA) of the chest showed subtle small bilateral filling defects within the pulmonary branches suggestive of bilateral pulmonary emboli (Figure 2).

Of note, the patient had been anticoagulated with warfarin since he was seven years old and has had three different inferior vena cava (IVC) filters placed to date; the first one was placed at age seven. Despite these measures, the patient has been hospitalized many times because of pulmonary embolism (PE). For his current hospitalization, he was treated with heparin drip (18 units/kg bolus followed by 1333 units/hour and adjusted based on hospital standardized protocol) and supportive care with intravenous fluid rehydration and pain control as needed was provided throughout his hospital stay. His home anticoagulation was switched from warfarin (4 mg daily) to apixaban (10 mg twice daily for seven days, followed by 2.5 mg twice daily) at time of discharge. He was asymptomatic at time of discharge. Prior to this current hospitalization, the patient had been hospitalized at our hospital twice over the past six months (for five days and four days) for pulmonary embolism. However, since the last discharge during which his anticoagulation was changed to apixaban (i.e., more than six months ago), the patient has not been seen at our hospital for complaints related to DVTs or PE.

## Discussion

We have presented a rare case of KTS with recurrent PE despite adequate anticoagulation with warfarin as evidenced by the therapeutic PT/INR values on presentation. This case highlights the difficulty that can be faced with patients with KTS in whom a high incidence of VTE exists owing to their anatomic abnormalities. In fact, it is well established that the incidence of VTE in KTS is between 14 and 22%, which is equal to or higher than the incidence associated with most other risk factors for VTE that have been integrated into clinical risk assessment scores such as the Wells score [5,7,8]. Although the importance of these clinical assessment scores cannot be undermined, their importance to at-risk patient populations, such as patients with KTS, may not be applicable. In such populations, suspicion for VTE should be high on the differential to warrant treatment even before definitive diagnostic studies are done. As highlighted in our case, even a therapeutic PT/INR value should not dissuade the physician from pursuing further VTE workup.

This is a rare case of KTS with recurrent VTE despite the achievement of therapeutic anticoagulation. It could be possible that other rare genetic causes of thrombophilia contributed to the recurrent PE seen in our patient. However, common causes of hypercoagulability such as protein C and S deficiencies, factor V Leiden, and heparin-induced thrombocytopenia were excluded. However, even in the presence of an occult genetic cause of hypercoagulability, a therapeutic PT/IRN of 37.3/3.2 should prevent clotting.

A similar case presentation, but with a poorer outcome, had earlier been described by Gianlupi et al. [9]. Although it can be difficult to make a direct comparison of their case and ours, it is of interest to highlight some important points. In their case, the patient had PE recurrence despite her INR of 2.31 on presentation. This contrasts with our patient whose INR was >3 on presentation. Thus, it is possible that the differences in INRs could have contributed to the outcomes observed. Also, in their patient, anticoagulation management was done with heparin and warfarin. Although the exact dose of heparin and warfarin used by them was unclear, the better outcome observed in our patient could have been, in part, due to the utilization of a new generation oral anticoagulation, apixaban, which was not available in the 1990s. This suggests that the new generation oral anticoagulation may be superior to warfarin in the management of thromboembolism in KTS patients.

Anticoagulation of patients with KTS can be challenging because they are prone to having AVM that can easily lead to bleeding. In our case, this was highlighted in that the patient had a history of chronic gastrointestinal bleed secondary to AVMs. Thus, the benefits of anticoagulation must be weighed with the risk of bleeding in such patients and, in certain cases, VTE prophylaxis may be preferably achieved with IVC filters rather than anticoagulation. Currently, there are no specific standard guidelines for anticoagulation prophylaxis in patients with KTS. This could be partly because of the rarity of the condition. However, developing such guidelines could prove helpful to clinicians.

## Conclusions

KTS is a rare congenital condition with associated VTE and PE complications that can be difficult to manage. We have presented a rare case of recurrent VTE and PE in a patient with KTS. His presentation is unique and highlights some important lessons: PE should remain on the top of differentials in KTS patients presenting with dyspnea even if initial laboratory findings suggest an alternate diagnosis; a therapeutic PT/INR laboratory value does not exclude VTE and PE, especially in a patient with KTS; risk-benefit of anticoagulation in patients with KTS must be weighed since AVMs are common in these patients and pose a bleeding risk, and delineating clear anticoagulation prophylaxis guidelines in patients with KTS will be helpful to clinicians.
